# The Impact of the COVID-19 Pandemic on the Registration and Care Provision of Mental Health Problems in General Practice: Registry-Based Study

**DOI:** 10.2196/43049

**Published:** 2023-03-10

**Authors:** Jan Vandamme, Simon Gabriël Beerten, Jonas Crèvecoeur, Steve Van den Bulck, Bert Aertgeerts, Nicolas Delvaux, Gijs Van Pottelbergh, Mieke Vermandere, Laura Tops, Thomas Neyens, Bert Vaes

**Affiliations:** 1 Academic Center for General Practice Department of Public Health and Primary Care KU Leuven Leuven Belgium; 2 Leuven Biostatistics and Statistical Bioinformatics Centre Faculty of Medicine KU Leuven Leuven Belgium; 3 Interuniversity Institute for Biostatistics and Statistical Bioinformatics Data Science Institute UHasselt Hasselt Belgium

**Keywords:** COVID-19, mental health, care provision, general practice, socioeconomic status

## Abstract

**Background:**

The impact of the COVID-19 pandemic on mental health in general practice remains uncertain. Several studies showed an increase in terms of mental health problems during the pandemic. In Belgium, especially during the first waves of the pandemic, access to general practice was limited. Specifically, it is unclear how this impacted not only the registration of mental health problems itself but also the care for patients with an existing mental health problem.

**Objective:**

This study aimed to know the impact of the COVID-19 pandemic on (1) the incidence of newly registered mental health problems and (2) the provision of care for patients with mental health problems in general practice, both using a pre–COVID-19 baseline.

**Methods:**

The prepandemic volume of provided care (*care provision*) for patients with mental health problems was compared to that from 2020-2021 by using INTEGO, a Belgian general practice morbidity registry. Care provision was defined as the total number of new registrations in a patient’s electronic medical record. Regression models evaluated the association of demographic factors and care provision in patients with mental health problems, both before and during the pandemic.

**Results:**

During the COVID-19 pandemic as compared to before the COVID-19 pandemic, the incidence of registered mental health problems showed a fluctuating course, with a sharp drop in registrations during the first wave. Registrations for depression and anxiety increased, whereas the incidence of registered eating disorders, substance abuse, and personality problems decreased. During the 5 COVID-19 waves, the overall incidence of registered mental health problems dropped during the wave and rose again when measures were relaxed. A relative rise of 8.7% and 40% in volume of provided care, specifically for patients with mental health problems, was seen during the first and second years of the COVID-19 pandemic, respectively. Care provision for patients with mental health problems was higher in older patients, male patients, patients living in center cities (*centrumsteden*), patients with lower socioeconomic status (SES), native Belgian patients, and patients with acute rather than chronic mental health problems. Compared to prepandemic care provision, a reduction of 10% was observed in people with a low SES.

**Conclusions:**

This study showed (1) a relative overall increase in the registrations of mental health problems in general practice and (2) an increase in care provision for patients with mental health problems in the first 2 years of the COVID-19 pandemic. Low SES remained a determining factor for more care provision, but care provision dropped significantly in people with mental health problems with a low SES. Our findings suggest that the pandemic in Belgium was also largely a “syndemic,” affecting different layers of the population disproportionately.

## Introduction

Similar to most European countries during the COVID-19 pandemic, Belgium has experienced multiple COVID-19 waves in the period from 2020-2021. Measures were taken to reduce the COVID-19 burden during these waves. The reduction of social contacts, self-isolation, and quarantine were the main nonpharmaceutical interventions [1].

Several studies showed an increase in mental distress during the pandemic [2-8]. This increase has, among others, been attributed to decreased social contact [9], financial uncertainty [10], and the fear of catching COVID-19 [11].

Surveys in Belgium showed a similar trend [12,13], as did multiple studies in the United Kingdom [14-16]. Interestingly, some studies showed a significant correlation between the incidence of depressive symptoms and the measures that were taken [17]. Government measures implemented during waves of infections tended to ease depressive symptoms [17].

During surges of COVID-19 infections, assessing mental health status in general practice was challenging. The imposed mitigation measures decreased access to routine care, and the increased burden of care for patients with COVID-19 left little room for treating other conditions such as mental health problems. Although some studies showed an increase in the number of anxiety-related visits [18], most studies reported a reduction in overall mental health consultations [19-22]. Additionally, there was evidence for reduced access to mental health care for patients with preexisting mental health problems [23].

Most studies on mental health during the COVID-19 pandemic were cross-sectional in nature and often showed a disparity between different nations and risk groups [24-26].

Thus, longitudinal data and a pre–COVID-19 baseline are often missing in international literature. These are important tools for countries to monitor their population’s mental health during a pandemic [27].

We aimed to fill this gap and provide a longitudinal overview of how the COVID-19 pandemic was experienced in Belgian general practice in terms of registered mental health problems (both during and before the pandemic) and what factors led to an increased or decreased volume of care for these patients.

Therefore, we evaluated the impact of the COVID-19 pandemic on (1) the registration of mental health problems and (2) care provision for patients with existing mental health problems in general practice. Our hypotheses were that the number of registrations of mental health problems would increase during the pandemic, relative to the years before, and that care provision for patients with existing mental health problems would drop, given the more limited access to care.

## Methods

### INTEGO

This was a retrospective cohort study using INTEGO, a general practice morbidity registry in Flanders, the Dutch-speaking region of Belgium [28]. The study methods have been described earlier, also in the context of COVID-19, and were adjusted for the current study [29]. The data from INTEGO is largely representative for the Flemish population [28].

Our study population was defined as all patients (of all ages) who visited 1 of the 105 INTEGO practices between February 1, 2018, and January 31, 2022, and consisted of 397,489 patients. Data were automatically collected via the electronic health record of the participating practices and included patient characteristics, medical history, and socioeconomic status (SES). Available demographic characteristics included the year of birth, sex, nationality (native Belgian or nonnative patients), and place of residence (postal code). Diagnoses were coded using the International Classification of Primary Care, second edition (ICPC-2). Low SES was defined as the presence of an increased reimbursement under the national health care insurance policy. In Belgium, patients effectively only pay a small, fixed amount of the total fees for medical care and medication (copay or *remgeld* in Dutch); the rest of the cost is reimbursed by health insurance. The reimbursement for patients with a low SES is higher, thus making this fixed amount lower.

### Collection of Data and Intervention Period

We evaluated the impact of the COVID-19 pandemic by (1) comparing the incidence of registered mental health diagnoses and (2) comparing care provision for patients with mental health problems 2 years before the pandemic (ie, February 1, 2018, to January 31, 2020) with the first 2 years of the pandemic (ie, February 1, 2020, to January 31, 2022). In this period, Belgium experienced 5 COVID-19 waves, approximately around the following dates: first wave (March 1 to May 31, 2020), second wave (October 1 to November 30, 2020), third wave (March 1 to May 31, 2021); fourth wave (October 1 to the end of 2021); and fifth (Omicron) wave (started December 2021) [30].

There was an issue with some data for care provision at the end of 2021 due to a data flow error from the electronic medical software to our database. This is indicated in the corresponding figure below.

### Outcome Measures

#### Registration of Mental Health Diagnoses

For the first part of the study, we examined the difference in the registration of incident mental health problems during and before the COVID-19 pandemic. “Incident” here means registered for the first time since the start of the study period.

All psychological ICPC-2 codes (P-codes) were categorized into 7 subgroups related to depression; anxiety; psychosis; eating problems, substance abuse, and personality problems; sexuality problems; sleeping problems; and other mental health problems. Eating problems, substance abuse, and personality problems were pooled because of the insufficiently high patient counts necessary for statistical analysis. For each of these subgroups, a separate analysis was carried out.

We then further distinguished between acute and chronic mental health problems based on the registered ICPC-2 code [31]. This information can be found in Table S1 in Multimedia Appendix 1.

#### Evolution of Care Provision

The second part of the study examined the evolution of care provision during the pandemic, compared to the 2 years before.

Care provision was defined as the number of new data entries added to one’s medical history over a predefined time window. These entries included medication prescriptions, laboratory test results, measurements of medical parameters, diagnoses, physical therapy referrals, and radiology orders. In the computation of care provision, multiple laboratory tests or medication prescriptions registered on the same date were counted as one entry.

Care provision was investigated in patients with an acute mental problem, patients with an incident chronic mental problem, and patients with a prevalent chronic mental problem. An acute mental problem was defined as an acute P-code that was present at the beginning of the defined period or incident during this period. An incident chronic mental problem described a chronic P-code that was newly registered during the defined period or had its initial registration no more than 16 weeks before this period. A prevalent chronic mental problem implied the registration of a chronic P-code that was made >16 weeks before the defined period.

### Statistical Analysis

Relative care provision was computed as the ratio between care provision in the first 2 years of the COVID-19 pandemic and care provision during the previous 2 years. More granular, biweekly care data were obtained by comparing the same calendar weeks within both periods. CIs for relative care provisions were computed by assuming a normal distribution for the logarithm of the relative care provision.

Generalized linear models analyzed care provision per patient with an existing mental health problem (ie, with a registered P-code) per year as a function of the covariates age, sex, nationality, center city, and SES, with the following reference categories as a basis for comparison in the model: aged 18-35 years, female, Belgian, living outside center cities, and high SES. Center cities (*centrumsteden*) are urban areas in Flanders with high population density and a central economic and cultural function in its surrounding area.

We compared care provision for the 3 predefined P-code groups as described above (acute, incident chronic, and prevalent chronic). Besides these demographic covariates, we included in the model the general practice that the patients visited to correct for registration differences across practices. We chose a fixed practice effect over a random effect as the distribution of these effects is strongly nonnormal and the high number of patients per practice allows for an accurate estimation. We modelled care provision using a Poisson distribution with log link. The model estimated the change in care provision for the reference category in the first and second years of the COVID-19 pandemic and the multiplicative effect of demographic covariates on care provision both in the period before the pandemic and during the first 2 years of the pandemic. This approach allowed us to assess the overall impact of the pandemic as well as how this impact differs for various demographic groups in the population. Statistical analysis was conducted in R statistical software (version 4.0.3; R Foundation for Statistical Computing).

### Ethical Approval

The INTEGO procedures were approved by the Ethics Committee Research UZ/KU Leuven (nr. ML1723) and by the Belgian National Privacy Commission’s Sectoral Committee (decision nr. 13.026 of March 19, 2013, last modified on April 17, 2018).

INTEGO was waived the need for individual informed consent, instead using an opt-out procedure for patients who do not want their data to be included in the database. This procedure was approved by the aforementioned ethical review board. There was no compensation or financial incentive provided for inclusion in the INTEGO database.

The procedures outlined in this study are in accordance with current Belgian guidelines and regulations. All study data were pseudonymized before use.

## Results

### Evolution of the Registration of Incident Acute and Chronic Mental Health Problems

#### General

**Figure 1:**
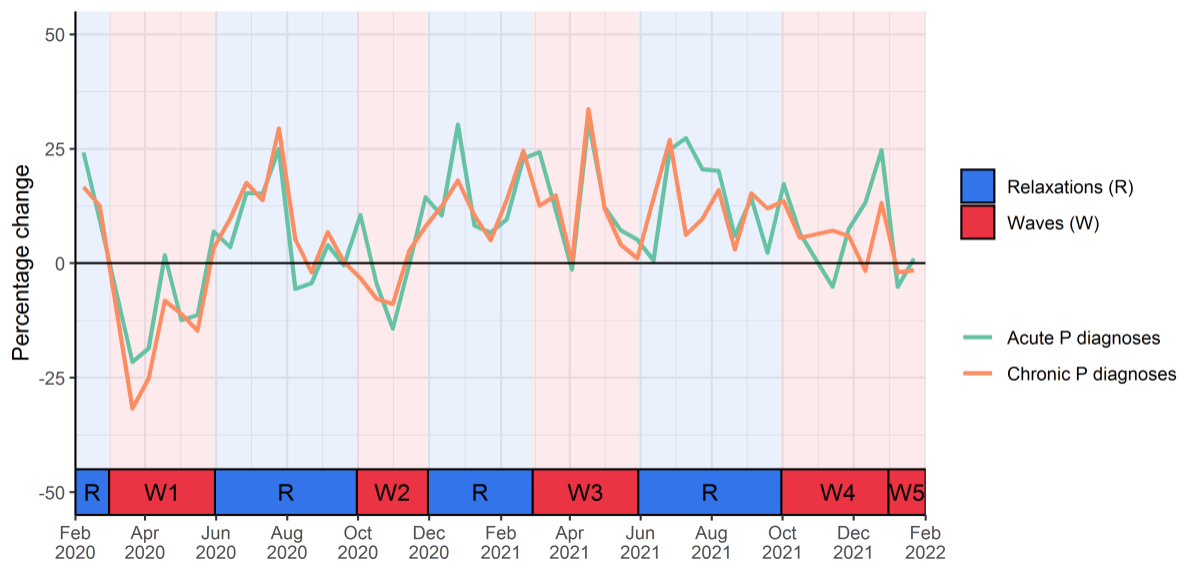
Evolution of incident acute and chronic mental health diagnoses (registered P-codes) during the first 2 years of the COVID-19 pandemic (February 1, 2020, to January 31, 2022) relative to the 2 previous years. P: psychological; P-code: psychological International Classification of Primary Care, second edition, code.

The incidence of registered acute and chronic mental health disorders showed a fluctuating course (Figure 1). This analysis is split into acute and chronic mental health diagnoses based on the ICPC-P codes (see Table S1 in Multimedia Appendix 1). Shortly before the first wave, the incidence of both acute and chronic mental health disorders was between 20% and 25% higher compared to the 2 years before, and this dropped to almost 25% less at the beginning of the first wave. When measures were relaxed, registrations rose again compared to the previous years until the end of July and normalized from August to October. During the second wave, the incidence of registered acute and chronic mental health disorders dropped again but less compared to the first wave. After the second wave, the incidence of registered disorders increased, especially for acute disorders. This fluctuating course became less pronounced in the second COVID-19 year, but especially for the acute disorders, the increase during relaxations can still be seen.

The evolution of the registration for the different subgroups during the study period can be seen in Figure S1 in Multimedia Appendix 1. The patient characteristics are summarized in Table S2 in Multimedia Appendix 1.

#### Mood Problems and Anxiety

During the first year of the COVID-19 pandemic, the registration of mood problems and anxiety increased significantly by 4% (95% CI 1%-8%) and 9% (95% CI 6%-13%), respectively (Table 1). During the first wave, the registration of anxiety rose significantly by 22% (95% CI 15%-30%). After the second wave, both mood problems and other mental health problems showed a significant incidence rise of 14% (95% CI 6%-22%) and 9% (95% CI 4%-14%).

For the second COVID-19 year, the results were similar. Registrations of anxiety and mood disorders rose by the same amount, although the incidence rates declined significantly around late 2021.

**Table 1:** The average number of registered mental health diagnoses per 1000 patient-years during the first and second year of the COVID-19 pandemic and percentage change in registered incidence relative to the 2 prepandemic years along with 95% CIs. Results are stratified by calendar period and P-code^a^ groups.

Group and year		February		March to May		June to September		October to November		December to January		Total	
		Mean	Percentage change (%; 95% CI)	Mean	Percentage change (%; 95% CI)	Mean	Percentage change (%; 95% CI)	Mean	Percentage change (%; 95% CI)	Mean	Percentage change (%; 95% CI)	Mean	Percentage change (%; 95% CI)
**Mood problems**													
	1	20.48	10 (0 to 22)	17.99	–11 (–16 to –5)	21.13	14 (8 to 20)	22.07	0 (–6 to 7)	25.14	14 (6 to 22)	20.78	4 (1 to 8)
	2	20.82	12 (1 to 24)	22.29	9 (3 to 15)	21.32	15 (9 to 21)	20.4	–2 (–8 to 3)	21.81	–12 (–20 to –3)	21	6 (3 to 9)
**Anxiety**													
	1	17.53	3 (–7 to 16)	21.28	22 (15 to 30)	18.38	11 (5 to 17)	18.63	0 (–8 to 7)	19.84	2 (–4 to 10)	19.04	9 (6 to 13)
	2	18.36	8 (–2 to 21)	20.59	19 (12 to 26)	18.49	12 (6 to 18)	16.8	–7 (–12 to –1)	18.7	–13 (–22 to –4)	18.35	5 (2 to 9)
**Psychosis**													
	1	2.36	19 (–13 to 63)	2.03	2 (–15 to 24)	2.33	20 (2 to 40)	2.38	9 (–11 to 36)	2.58	0 (–18 to 22)	2.3	9 (0 to 20)
	2	2.15	9 (–20 to 50)	2.23	12 (–5 to 35)	2.27	17 (0 to 37)	2.16	–2 (–18 to 16)	2.26	–21 (–41 to 5)	2.22	6 (–3 to 15)
**Sleeping problems**													
	1	15.39	22 (8 to 38)	10.99	–16 (–23 to –9)	13.95	4 (–2 to 10)	15.25	–5 (–12 to 3)	17.99	5 (–2 to 14)	14.07	0 (–4 to 2)
	2	14.28	14 (0 to 29)	14.8	11 (4 to 20)	14.49	8 (2 to 15)	13.71	–9 (–15 to –2)	16.01	–22 (–30 to –13)	14.33	1 (–2 to 4)
**Eating problems, substance abuse, and personality problems**													
	1	7.05	6 (–11 to 27)	4.47	–33 (–41 to –24)	5.79	–20 (–27 to –11)	6.91	–10 (–21 to 1)	6.8	–13 (–23 to –1)	5.89	–18 (–22 to –13)
	2	5.79	–12 (–27 to 6)	6.61	0 (–10 to 9)	6.42	–10 (–18 to –2)	5.15	–30 (–37 to –21)	5.93	–33 (–44 to –20)	6.03	–15 (–20 to –11)
**Sexuality problems**													
	1	2.27	–4 (–29 to 30)	2.57	15 (–2 to 37)	2.84	15 (0 to 33)	2.8	–4 (–21 to 16)	2.9	1 (–16 to 23)	2.72	7 (0 to 16)
	2	3	26 (–3 to 66)	2.63	19 (0 to 40)	2.61	6 (–7 to 23)	2.56	–8 (–22 to 7)	2.79	–10 (–31 to 16)	2.65	4 (–3 to 13)
**Other mental health problems**													
	1	45.28	23 (14 to 33)	32.18	–21 (–24 to –17)	39.66	9 (5 to 13)	46.38	–4 (–9 to 0)	48.89	9 (4 to 14)	39.66	0 (–2 to 2)
	2	47.32	29 (20 to 39)	49.6	21 (17 to 26)	45.25	25 (20 to 29)	44.38	–1 (–5 to 2)	47.6	–6 (–12 to 0)	44.92	13 (11 to 15)

^a^P-code: psychological International Classification of Primary Care, second edition, code.

#### Eating Disorders, Substance Abuse, and Personality Problems

The registration of eating problems, substance abuse, and personality problems showed a significant decrease of 18% (95% CI 13%-22%) in the first COVID-19 year and a decrease of 15% (95% CI 11%-20%) in the second year. During these 2 years, there were no significant fluctuations but rather a steady decrease in registrations each period, regardless of wave.

#### Psychosis, Sexuality, and Sleeping Problems

The incidence of registered psychosis and sexuality problems increased nonsignificantly during both the first and second COVID-19 years. The month before the first wave was characterized by an increased registration for all groups, except sexuality problems. The registration of sleeping problems then was 22% (95% CI 8%-35%) higher compared to the same period in the 2 previous years.

### Evolution of Care Provision for Patients With Mental Health Problems and the Effect of Demographic Factors

**Figure 2:**
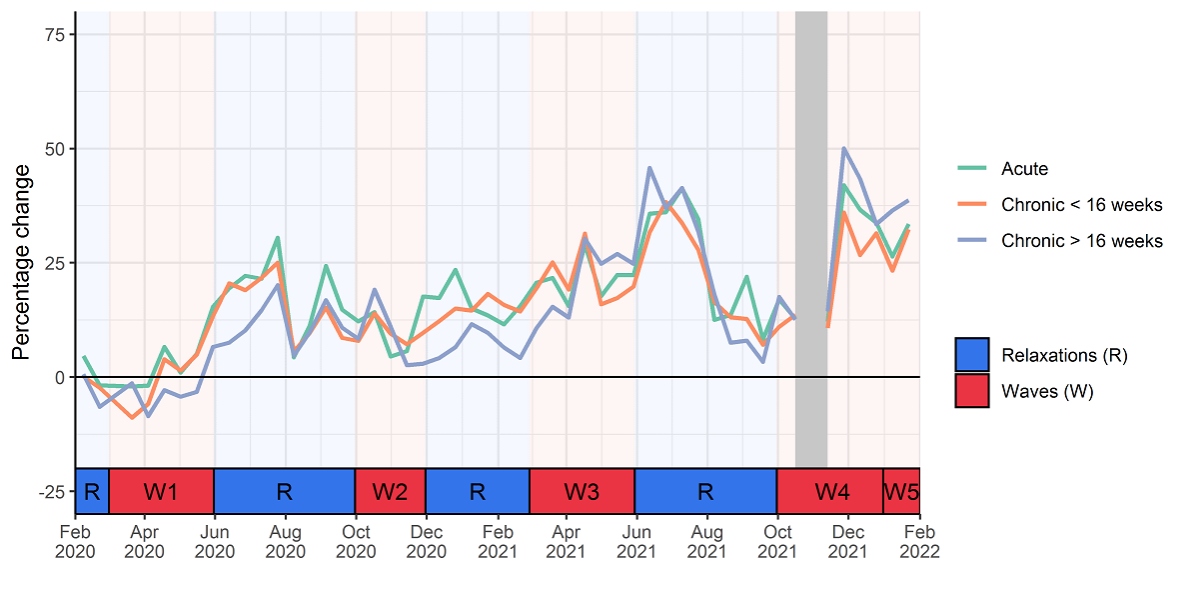
Evolution of care provision for patients with a registered acute, incident chronic, and prevalent chronic mental problem during the first 2 years of the COVID-19 pandemic (February 1, 2020, to January 31, 2022) relative to the 2 years before. Gray bar: no data due to technical issues with data flow.

Before the start of the pandemic, almost no difference in care provision for the 3 groups (acute, incident chronic, and prevalent chronic mental health problems) was observed compared to the 2 years before COVID-19. In this analysis, patients with a recent chronic P-code diagnosis were split from those who received their diagnoses longer ago to see whether additional care was mainly given close to the onset of diagnosis or whether older chronic P-code diagnoses also resulted in continued care. Care provision almost stayed the same at the start of the first wave, but provision rose for all 3 groups during the first relaxation period. Starting from the end of May 2020, overall care provision remained higher than in previous years (Figure 2).

In general, there was 8.7% (95% CI 8.4%-9.1%) more care provided to patients in the reference category during the first year of COVID-19 compared to the 2 years before the pandemic. In the second year, this increased to 38.3% (95% CI 37.2%-39.5%).

**Figure 3:**
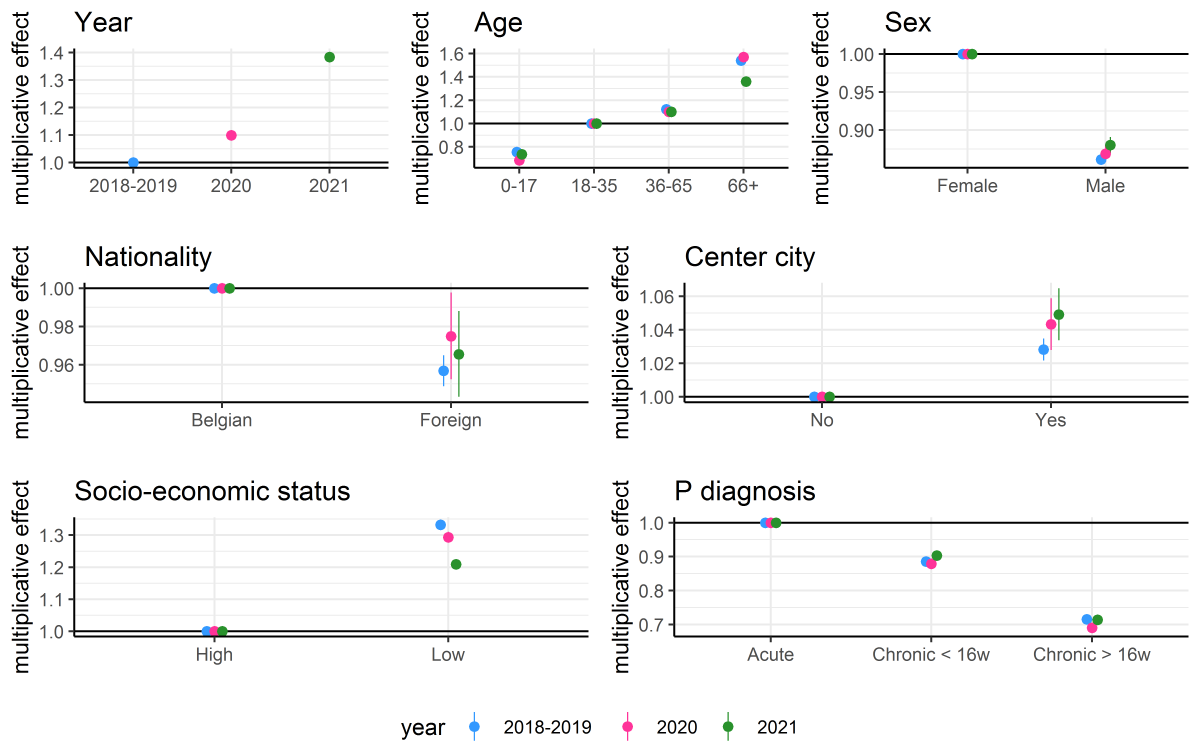
Multiplicative effect and associated 95% CIs of covariates on care provision for mental health problems per patient per year. Separate parameters were estimated for the period February 1, 2018, to January 31, 2020, and the period including the 2 COVID-19 years (February 1, 2020, to January 31, 2022), which are labelled 2018-2019, 2020 and 2021, respectively.

In general, more care was provided for acute mental health problems and in older patients, female patients, native Belgian patients, patients living in center cities, and patients with a low SES (Figure 3).

Care provision for foreign patients somewhat increased compared to the 2 years before COVID-19 (although lower in 2021 than in 2020) but stayed slightly less than native Belgian patients. In patients with a low SES, we observed more care provision than for those with a high SES during the study period, although care provision dropped 10% for people with a low SES during the pandemic compared to the 2 years before.

Compared to patients with acute mental health problems, patients with incident chronic and prevalent chronic mental health problems had less care provision (around 10% and 30% less, respectively). This was similar to the period before COVID-19.

## Discussion

### Principal Findings

This registry-based study showed that the first 2 years of the COVID-19 pandemic had significant repercussions on the registration of new mental health problems and care provision for patients with mental health problems in general practice.

Waves of infections resulted in a drop in registered mental health problems and an increase of up to 25% during relaxation periods when compared to the previous years. This phenomenon was less pronounced in the second COVID-19 year.

With a rise of around 9% in the first COVID-19 year and almost 40% in the second year, overall care provision for patients with mental health problems increased during the first 2 years of the pandemic. In general, care provision was 50% higher among those aged 66 years and over, around 4% higher for patients living in center cities, and approximately 30% higher for those with a lower SES; it was lower among male (–10%) and nonnative Belgian (–4%) patients. Patients with acute mental health problems had the most care provided to them with 10% to 30% more registrations than patients with chronic mental health problems.

During the pandemic, care provision dropped by 10% in patients with a lower SES.

### Comparison With Literature

There currently is little evidence on the care provision for patients with preexisting mental health problems during COVID-19, especially in general practice [23]. A similar UK-based primary care cohort study showed the same overall drop in the registration of mental health problems at the start of the pandemic and the same normalization of these rates after April 2020 [19]. We extended this study period and saw a continuing relative increase in registrations of new mental health problems during the pandemic as compared to before. This increase was fluctuating and corresponded to the specific COVID-19 waves in Belgium.

The multivariate analysis for our definition of care provision that we conducted in this study was new. We provided more evidence to the hypothesis that low SES affected the outcomes disproportionately, despite these patients having the highest recorded diagnoses of depression, anxiety, and self-harm before the pandemic [19]. The overall increase in registered mood problems and anxiety matched the rise in mental distress seen in surveys [12,14-16].

Annual reports of Sciensano (Belgium’s national public health institute) and Belgium’s Superior Health Council signaled a high prevalence of sleep disturbance, anxiety, and depressive disorders during the COVID-19 pandemic [12,30,32]. Our finding that the registration of mood disorders rose when strict measures were taken is confirmed by the report of the Superior Health Council [32]. The impact was more pronounced during stringent or long-lasting measures, for instance, due to the existing uncertainty and lack of social interaction. However, during waves with high viral spread, it is not inconceivable that general practitioners were overwhelmed by COVID-19–related care, leaving little room for other clinical work. This could have led to an underestimation of the actual burden of mental health problems during such periods.

Additionally, both Belgian reports also noted different groups being at risk for more mental health problems, such as adolescents, single individuals, people entitled to social security payments, people with low SES, or people with known mental health problems [12,32], which was in keeping with our findings.

Interestingly, we found that care provision for people with lower SES *decreased* during the pandemic.

### Strengths and Limitations

The main strength of this study was the use of longitudinal data from a large general practice registry, which allowed us to investigate a large sample of the Flemish population. We calculated a proxy for the SES of the patients in INTEGO, and the correlation of this social determinant could be further assessed. Since INTEGO weekly and automatically collects data via the electronic health record of the participating general practices, further monitoring is easily accessible.

There are however some limitations to this study. Data were collected from 105 practices that are individually responsible for delivering qualitative and complete data. Possible registration errors were minimized by accounting for interpractice differences, and steps have been taken to improve data completeness [33]. However, there might be a general underregistration of mental health problems in general practice (especially subclinical problems) that we were unable to pick up due to them not being coded.

Our figures for registrations of mental health problems, especially during the first wave, might be underestimations because of patients simply not visiting general practices due to a fear of COVID-19 in general.

Furthermore, we defined care provision as the number of contacts added to a patients’ medical record, but this does not necessarily reflect the quality of each contact in regard to the depth of care provision. Additionally, a decrease in care provision does not necessarily imply a proportional change in the content of care (eg, a decrease might be a good thing if there are fewer unnecessary prescriptions but a bad thing if it leads to fewer referrals to specialized care for more severe problems). Finally, although the grouping of P-codes was done after consensus, the classification may be somewhat arbitrary, since some mental health problems could not be studied separately, as we opted to pool them due to insufficiently high patient counts.

It should also be noted that teleconsultations may be a useful and viable alternative to a live consultation for mental health problems in times of pandemic [34]. Our data could not distinguish between a live consultation and a teleconsultation.

### Implications for Practice and Future Research

Obviously, the measures taken in the first 2 years of the COVID-19 pandemic have had a noticeable effect on mental health and care provision for mental health problems in Flanders. The negative impact may also have been compounded by what the Superior Health Council labelled as the “infodemic”—in which negative topics about COVID-19 were disseminated in an unmotivated and almost autocratic way [32]. Governmental policies with a commitment to improve nationwide resilience and social security, using efficient and accessible health care resources, must be a priority [35]. It is clear that communication about the pandemic must be given clearly and at the right time, particularly to the most vulnerable groups of the population [32].

The decreasing trend in care provision for socioeconomically vulnerable patients during the COVID-19 pandemic is a notable finding, deserving of further study. Similar to the infodemic discussed earlier, COVID-19 has also been regarded by some experts as a “syndemic” [36,37]. A syndemic in this context refers to a pandemic exacerbated by—previously disregarded or undervalued—social determinants of health: housing, income, neighborhood deprivation, and other social risk factors for disease. Although COVID-19 might not be regarded as a syndemic everywhere in the world [38], based on our results, we would argue that this might be the case in Belgium.

### Conclusions

Our study found that the incidence of new registrations for mental health problems was generally higher during the COVID-19 pandemic than in the 2 years before. There was a slight decrease during the first wave, after which the incidence continued to climb, following a fluctuating course.

In addition, we studied care provision for patients with established mental health problems in general practice during the first 2 years of the COVID-19 pandemic, compared to the 2 years before. Care provision was generally higher during the pandemic than before. Low SES remained a determining factor for more care provision, but care provision dropped significantly in people with mental health problems with a low SES.

Based on our findings, we would suggest that the pandemic in Belgium was also largely a “syndemic,” affecting different layers of the population disproportionately.

## Appendix

Multimedia Appendix 1 The list of ICPC-2 groups, evolution of registered P-codes, and distribution of patient characteristics. ICPC-2: International Classification of Primary Care, second edition; P-code: psychological ICPC-2 code.

## Abbreviations

ICPC-2: International Classification of Primary Care, second edition
P-code: psychological International Classification of Primary Care, second edition, code
SES: socioeconomic status

## Data Availability

The data sets generated and analyzed during this study are not publicly available due to the inclusion of protected health information but can be made available after deidentification upon reasonable request to the corresponding author (SGB).
