# Supplementary material for: The Impact of the COVID-19 Pandemic on the Registration and Care Provision of Mental Health Problems in General Practice: Registry-Based Study
Source: JMIR Public Health Surveill. 2023 Mar 10;9:e43049. doi: 10.2196/43049 (PMC10039400; doi:10.2196/43049)
Supplement: Multimedia Appendix 1 [file publichealth_v9i1e43049_app1.pdf]

Table S1: List of ICPC-2 groups

GROUP	P-CODE	DEFINITION	ACUTE - CHRONIC
Mood	P03	Feeling depressed	A
	P04	Feeling/behaving irritable/angry	A
	P05	Senility, feeling/behaving old	A
	P76	Depressive disorder	C
	P77	Suicide/suicide attempt	A
Anxiety	P01	Feeling anxious/nervous/tense	A
	P02	Acute stress reaction	A
	P27	Fear of mental disorder	A
	P74	Anxiety disorder/anxiety state	C
	P79	Phobia/compulsive disorder	C
	P82	Post-traumatic stress disorder	C
Psychosis	P71	Organic psychosis other	C
	P72	Schizophrenia	C
	P73	Affective psychosis	C
	P98	Psychosis NOS/other	C
Eating	P11	Eating problem in child	C
Substance abuse	P86	Anorexia nervosa/bulimia	C
Personality disorder	P15	Chronic alcohol abuse	C
	P16	Acute alcohol abuse	C
	P17	Tobacco abuse	C
	P18	Medication abuse	C
	P19	Drug abuse	C
	P80	Personality disorder	C
Sexuality	P07	Sexual desire reduced	A
	P08	Sexual fulfilment reduced	A
	P09	Sexual preference concern	A
Sleeping disorder	P06	Sleeping disorder	A
Other	P12	Enuresis	A
	P22	Child behavior symptom	A
	P23	Adolescent behavior symptom	A
	P24	Specific learning problem	A
	P25	Phase of life problem adult	A
	P10	Stammering/stuttering/tic	A
	P20	Memory disturbance	A
	P28	Limited function/disability	C
	P29	Psychological symptom other	A
	P70	Dementia	C

	P75<br>P78<br>P81<br>P85<br>P99	Somatization disorder<br>Neurasthenia<br>Hyperkinetic disorder<br>Mental retardation<br>Psychological disorders other	C<br>A<br>A<br>C<br>A

Evolution of registered P-codes

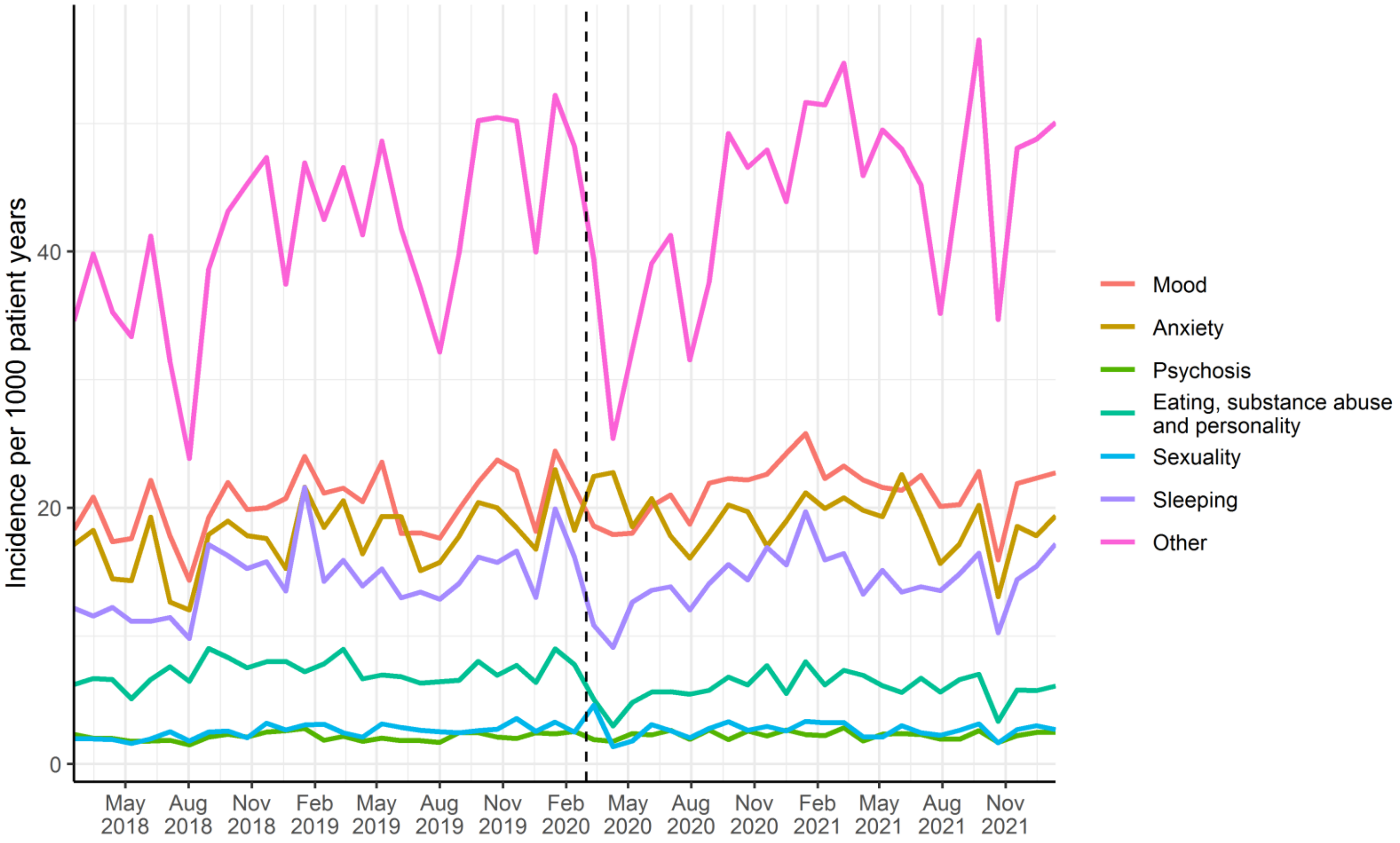

Figure S1: Evolution of registered P-codes grouped by relevance during the first two COVID-19 years and the two years before COVID-19 (February 1, 2018 – January 15, 2022). The vertical gray dashed line indicates the start of the COVID-19 pandemic in Belgium.

Note: The category 'Other' consists mainly of P29 ("Psychological symptom/complaint other") and P78 ("Neurasthenia") and is in practice most often used to code 'occupational burnout', which has no corresponding ICPC code.

Table S2: Distribution of patient characteristics for the years 2018-2021 in the data

**Characteristic**	**Outcome**	**2018**	**2019**	**2020**	**2021**
**Age**	Missing	99	116	121	302
	18-	3109	3679	3951	4657
	18-35	11024	12441	12681	14035
	36-65	21718	24104	24946	26889
	65+	8931	10376	11550	12330
**Sex**	Missing	126	146	152	346
	Female	25477	28700	30250	32840
	Male	19278	21870	22847	25027
**Nationality**	Missing	6154	6446	6706	7618
	Belgian	36048	41082	43147	46734
	Foreign	2679	3188	3396	3861
**Center city**	No	30224	34186	36087	39504
	Yes	14657	16530	17162	18709
**Socioeconomic status**	Missing	415	497	559	795
	High	33981	38706	40817	44753
	Low	10485	11513	11873	12665
